# Colour of sputum is a marker for bacterial colonisation in chronic obstructive pulmonary disease

**DOI:** 10.1186/1465-9921-11-58

**Published:** 2010-05-14

**Authors:** Marc Miravitlles, Alicia Marín, Eduard Monsó, Sara Vilà, Cristian de la Roza, Ramona Hervás, Cristina Esquinas, Marian García, Laura Millares, Josep Morera, Antoni Torres

**Affiliations:** 1 Fundació Clínic. Institut D'Investigacions Biomèdiques August Pi i Sunyer (IDIBAPS). Ciber de Enfermedades Respiratorias (CIBERES), Barcelona, Spain; 2 Department of Pneumology, Hospital Germans Trias i Pujol. Autonomous University of Barcelona; Ciber de Enfermedades Respiratorias (CIBERES), Barcelona, Spain; 3 Department of Pneumology, Hospital Germans Trias i Pujol, Ciber de Enfermedades Respiratorias (CIBERES), Badalona, Barcelona, Spain; 4 Medical Department, Bayer Schering Pharma, Sant Joan Despi, Barcelona, Spain; 5 Department of Pneumology, Institut Clínic del Tòrax (IDIBAPS), Hospital Clínic, Ciber de Enfermedades Respiratorias (CIBERES), Barcelona, Spain

## Abstract

**Background:**

Bacterial colonisation in chronic obstructive pulmonary disease (COPD) contributes to airway inflammation and modulates exacerbations. We assessed risk factors for bacterial colonisation in COPD.

**Methods:**

Patients with stable COPD consecutively recruited over 1 year gave consent to provide a sputum sample for microbiologic analysis. Bronchial colonisation by potentially pathogenic microorganisms (PPMs) was defined as the isolation of PPMs at concentrations of ≥10^2 ^colony-forming units (CFU)/mL on quantitative bacterial culture. Colonised patients were divided into high (>10^5 ^CFU/mL) or low (<10^5 ^CFU/mL) bacterial load.

**Results:**

A total of 119 patients (92.5% men, mean age 68 years, mean forced expiratory volume in one second [FEV_1_] [% predicted] 46.4%) were evaluated. Bacterial colonisation was demonstrated in 58 (48.7%) patients. Patients with and without bacterial colonisation showed significant differences in smoking history, cough, dyspnoea, COPD exacerbations and hospitalisations in the previous year, and sputum colour. Thirty-six patients (62% of those colonised) had a high bacterial load. More than 80% of the sputum samples with a dark yellow or greenish colour yielded PPMs in culture. In contrast, only 5.9% of white and 44.7% of light yellow sputum samples were positive (*P *< 0.001). Multivariate analysis showed an increased degree of dyspnoea (odds ratio [OR] = 2.63, 95% confidence interval [CI] 1.53-5.09, *P *= 0.004) and a darker sputum colour (OR = 4.11, 95% CI 2.30-7.29, *P *< 0.001) as factors associated with the presence of PPMs in sputum.

**Conclusions:**

Almost half of our population of ambulatory moderate to very severe COPD patients were colonised with PPMs. Patients colonised present more severe dyspnoea, and a darker colour of sputum allows identification of individuals more likely to be colonised.

## Background

Exacerbations are the main cost driver in chronic obstructive pulmonary disease (COPD), have a negative impact on the clinical course of the patients and are associated with increased mortality [1-3]. Around 70% of exacerbations are infectious in nature, either bacterial, viral or mixed [4-7]. It has been shown that airway bacterial load in the stable state contributes to airway inflammation and modulates the character and frequency of exacerbations [8,9]. There is also evidence that bronchial colonisation influences the decline in lung function over time [10]. Different studies in which respiratory samples were obtained by the protected specimen brush (PSB) technique have shown a high prevalence of bronchial colonisation in COPD patients [5,11,12]. However, the practice of bronchoscopy to assess bronchial colonisation in routine clinical practice is not feasible and data that support the use of sputum samples to identify patients colonised by potentially pathogenic microorganisms (PPMs) are required.

Consequently, a cross-sectional study was designed to assess the frequency of bronchial bacterial colonisation using sputum samples and to identify risk factors for colonisation in stable ambulatory patients with COPD. The clinical characteristics of patients colonised and non-colonised with PPMs were compared as were those of patients with low and high bacterial loads in sputum samples.

## Methods

A cross-sectional study was carried out to assess clinical characteristics associated with bronchial colonisation in stable ambulatory COPD patients. These patients were visited at the outpatient respiratory clinics of two acute-care tertiary hospitals in Barcelona, Spain and were consecutively recruited over one year. After completing the collection of data for this study, patients with bronchial colonisation were included in a randomised trial of antibiotic treatment the results of which have been reported elsewhere [13]. The protocol was approved by the institutional review board and all patients gave written informed consent.

### Study population

Eligible patients were adults over 40 years of age, smokers or ex-smokers of at least 10 pack-years, with stable COPD, defined as a post-bronchodilator forced expiratory volume in one second (FEV_1_)/forced vital capacity (FVC) ratio of <70%. A FEV_1 _of <60% of the predicted value higher than 0.70 litres and a negative bronchodilator test (increase in FEV_1 _<200 mL and <12% of baseline) was required for inclusion in the study as was a history of at least one documented exacerbation in the previous year. Clinical stability was defined by the attending physician on clinical grounds based on the absence of symptoms of exacerbation and use of any oral or systemic antibiotics or a course of oral corticosteroids in the 6 weeks prior to inclusion.

The exclusion criteria were the following: (1) previous diagnosis of bronchial asthma, bronchiectasis demonstrated by a chest X-ray or computed tomography (CT) scan, or other relevant pulmonary diseases apart from COPD; (2) chronic treatment with oral corticosteroids at any dose; (3) formal contraindication for sputum induction or impossibility to obtain a valid sputum sample for analysis; and (4) participation in another clinical study concurrently or within the previous 3 months.

### Study procedures

At the time of inclusion in the study, the investigator verified that the patient met the eligibility criteria and details of medical history were recorded. Information regarding comorbidities, particularly cardiovascular diseases, diabetes and liver or renal failure was collected. A forced spirometry was performed following criteria of the Spanish Society of Pneumology and Thoracic Surgery [14] and sputum samples were obtained. Patients unable to produce sputum were susceptible to reassessment for airway colonisation at least one month after the initial investigation for a maximum of three consecutive visits.

### Microbiological sputum study

A sputum sample was obtained and processed within 60 minutes on the day of the visit according to standard methods [13,15,16]. Patients who did not produce sputum spontaneously underwent sputum induction. In brief, patients were pretreated with an inhaled β_2_-agonist ten minutes before the nebulisation of isotonic saline (0.9%) with an ultrasonic nebuliser (Ultraneb2000, DeVilbiss Healthcare Inc., Somerset, PA, USA), that was followed by increasing concentrations of hypertonic saline (3%, 4% and 5%), for 7 min with each concentration. After every induction, the patient attempted to obtain a sputum sample by coughing, and the nebulisation procedure was stopped when the sputum volume collected was 1 mL or more [17]. In current smokers, sputum induction was performed after at least 6 hours of tobacco abstinence. The purulence of sputum was graded in a scale from 1 to 5 according to the colour from white -1- to greenish -5-, always by the same researcher at each centre. The sample was weighed and processed with a 4-fold volume of dithiothreitol (Sputasol, Oxoid Ltd., Hants, UK) and was cultured. Sputum samples were serially diluted and plated on chocolate agar enriched, chocolate agar with bacitracin, *Haemophilus*-selective agar, blood agar, and McConkey agar. Plates were incubated for 24-48 hours at 37°C and in 5% CO_2 _atmosphere. Microorganisms were identified by colony morphology, Gram staining and specific culture conditions (e.g., requirements for factors for growth, presence of oxidase and catalase, porphyrin synthesis). Cultures were considered positive for bronchial colonisation if microorganisms considered as PPMs such as *Haemophilus influenzae, Haemophilus parainfluenzae, Streptococcus pneumoniae, Moraxella catarrhalis, Pseudomonas aeruginosa*, enterobacteria and/or *Staphylococcus aureus *were grown at loads of at least 100 colony-forming units (CFU)/mL according to previously defined criteria [18,19]. Colonised patients were then divided into high (>10^5 ^CFU/mL) or low (≤10^5 ^CFU/mL) bacterial load according to previous studies [4,8].

Sputum concentrations of pro-inflammatory cytokines, including interleukin-1 (IL-1), interleukin-6 (IL-6), interleukin-8 (IL-8), and tumour necrosis factor-alpha (TNF-alpha) were measured using quantitative sandwich immunoassay techniques in processed supernatants as previously described [20].

### Statistical analysis

Variables were presented as mean values and standard deviations, those not following a normal distribution were presented as median and interquartile range (IQR, 25th-75th percentile). Categorical variables were compared with the chi-square test and continuous variables with the Student's *t *test or the Mann-Whitney U test when data departed from normality. Following univariate analysis, variables were included in two stepwise logistic regression models constructed as exploratory analysis to identify independent risk factors for bronchial colonisation and factors significantly associated with high bacterial load as opposed to low bacterial load and sterile sputum cultures. The variables included in the models were: age, gender, active versus ex-smoker, pack-years of smoking, FEV_1 _(% predicted), degree of dyspnoea, colour of sputum, cardiovascular comorbidity and number of exacerbations and hospitalisations the previous year. Bilateral two-tailed hypotheses were formulated and 95% confidence intervals (CI) were calculated. Statistical significance was set at *P *< 0.05.

## Results

A total of 119 patients (92.5% men) with a mean (standard deviation, SD) age of 68.1 (9.1) years were studied. The clinical characteristics of these patients are reported in Table 1. Induction of sputum was necessary to obtain a valid sputum sample in only 5 cases (3 in one centre and 2 in the other). Bacterial colonisation was demonstrated in 58 (48.7%) patients, 2 in samples obtained by sputum induction. Results of sputum microbiology are shown in Table 2. Colonisation by a single PPM was recorded in 50 patients. Eight subjects yielded more than one PPM in their sputum. *Haemophilus influenzae *and *H. parainfluenzae *made up 72% of all bacterial isolates.

**Table 1:** Clinical characteristics of the study population

Data	Frequency
Subjects, no.	119
Sex, men, no. (%)	112 (92.5)
Age, years, mean (SD)	68.1 (9.1)
Current smokers, no. (%)	11 (9.2)
Smoking, pack-years, mean (SD)	40 (21.1)
Cardiovascular morbidity, no. (%)	36 (29.7)
Exacerbations in the previous year, mean (SD)	1.3 (0.5)
Requiring hospital admission	0.3 (0.5)
Post-bronchodilator spirometry, mean (SD)	
FVC, mL	2790 (942)
FVC, %	68.9 (19.2)
FEV_1_, mL	1406 (493)
FEV_1_, %	46.4 (14.1)

**Table 2:** Potentially Pathogenic Microorganisms (PPMs) isolated in colonised COPD patients.

	No. (%)
Microorganisms isolated	
*Haemophilus influenzae*	21 (42)
*Haemophilus parainfluenzae*	15 (30)
*Pseudomonas aeruginosa*	5 (10)
*Streptococcus pneumoniae*	4 (8)
*Moraxella catarrhalis*	4 (8)
*Staphylococcus aureus*	1 (2)
	
Mixed colonisations (from the above microorganisms)	
*H. influenzae *+ *S. pneumoniae*	1
*H. influenzae *+ *P. aeruginosa*	3
*H. influenzae *+ *H. parainfluenzae*	2
*P. aeruginosa *+ *S. viridans*	2

There were significant differences in cigarette consumption, cough, dyspnoea, comorbidities, COPD exacerbations and hospitalisations in the previous year, and sputum colour between patients with and without bacterial colonisation (Table 3).

**Table 3:** Differences between stable COPD patients with and without bacterial colonisation

Variables	Colonised (n = 58)	Not colonised (n = 61)	*P *value
Sex, men, no. (%)	54 (93.1)	55 (90.2)	0.74
Age, years, mean (SD)	68.3 (8.3)	67.6 (9.8)	0.67
Current smokers, no. (%)	7 (12.1)	4 (6.6)	0.35
Smoking, pack-years, mean (SD)	46.7 (25.1)	34.2 (23.4)	0.006
Cardiovascular morbidity, no. (%)	22 (37.9)	20 (32.8)	0.23
Comorbid conditions, mean (SD)	1.06 (0.99)	0.61 (1.02)	0.025
Use of inhaled steroids, no. (%)	46 (79.3)	47 (77.1)	0.83
Symptoms, no. (%)			
Dyspnoea	56 (96.5)	58 (95.1)	0.72
Cough	44 (75.9)	56 (91.8)	0.024
Expectoration	57 (98.3)	58 (95.1)	0.62
Grade of dyspnoea, mean (SD)	1.78 (0.92)	1.15 (0.54)	<0.001
Exacerbations in the previous year, no. (%)			
Number			0.021
≤2	31 (53.4)	47 (77.1)	
>2	27 (46.6)	14 (22.9)	
Requiring hospital admission			0.007
None	36 (62.1)	51 (83.6)	
≤1	16 (27.6)	10 (16.4)	
>1	6 (10.3)	0	
Lung function tests, mean (SD)			
FVC, mL	2852.7 (979.1)	2710.9 (911.5)	0.41
FVC, %	70.5 (19.5)	66.7 (18.9)	0.28
FEV_1_, mL	1411.4 (511.7)	1380.0 (433.1)	0.72
FEV_1_, %	47.4 (15.2)	45.1 (13.1)	0.38
FEV_1_/FVC	50.4 (11.8)	52.6 (13.9)	0.43
Sputum analysis			
Colour, mean (SD)	2.94 (1.0)	1.56 (0.8)	<0.001
Pro-inflammatory cytokines, median (IQR) in pg/mL			
IL-1, n = 53	14 (4-432)	168 (49-758)	0.82
IL-6, n = 53	258 (76-653)	112 (33-368)	0.62
IL-8, n = 61	13480 (1335-43400)	5390 (252-14335)	0.27
TNF-alpha, n = 54	45 (20-94)	35 (10-183)	0.12

FVC = forced vital capacity; FEV_1 _= forced expiratory volume in the first second; IQR = interquartile range; IL= interleukin; TNF = tumour necrosis factor.

**Figure 1:**
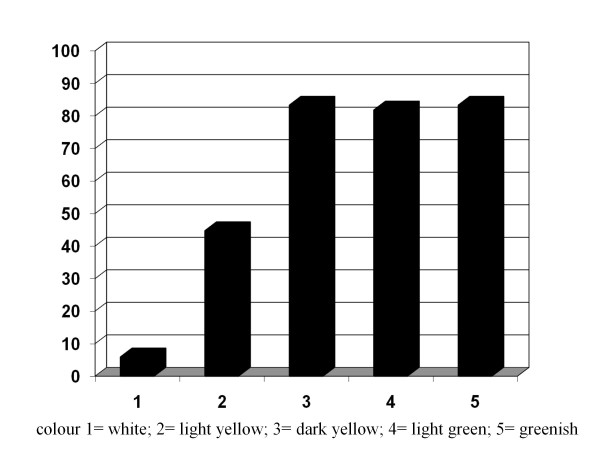
**Percentage of bacterial colonisation according to sputum colour (differences statistically significant at *P *< 0.001)**.

The distribution of colonised patients according to sputum colour is presented in Figure 1. Samples with colour 1 (white) were predominantly sterile, whereas in the samples with colours 3 to 5 (yellow to greenish) the prevalence of colonisation was higher than 80%. Colour number two (light yellow) was not discriminative between colonised and non-colonised.

When colonised patients were divided according to bacterial load, 36 patients had a high bacterial load (>10^5 ^CFU/mL) and the remaining 22 had a low bacterial load (≤10^5 ^CFU/mL). The characteristics of colonised patients with a high bacterial load (n = 36) were compared with a group formed by non-colonised patients (n = 61) and those with a low bacterial load (n = 22) considered together (n = 83). Statistically significant differences between the two groups in smoking (pack-years), cough, grade of dyspnoea, hospitalisations in the previous year and sputum colour persisted when patients with high bacterial loads were compared with the remaining patients (Table 4). Sufficient sputum for inflammatory analysis was available from only 61 subjects, all from spontaneous sputum. Sputum concentrations of inflammatory markers showed a great inter-individual variability and did not follow a normal distribution. There were no significant differences in sputum concentrations for any of the inflammatory markers analysed between patients with or without bacterial colonisation (Table 3). The lack of significance persisted when patients with high bacterial load were compared with those with low bacterial load and not colonised. However, in this last comparison, patients with high bacterial load presented consistently (but not significantly) higher concentrations of all pro-inflammatory cytokines except IL-6 (Table 4).

**Table 4:** Differences between colonised and non-colonised COPD patients according to bacterial load

Variables	**High bacterial load (≥10**^**5**^**) (n = 36)**	**Low bacterial load (<10**^**5**^**) and not colonised (n = 83)**	*P *value
Sex, men, no. (%)	33 (91.7)	76 (91.6)	0.98
Age, years, mean (SD)	68.6 (6.9)	67.7 (9.9)	0.63
Current smokers, no. (%)	6 (16.7)	5 (6)	0.086
Smoking, pack-years, mean (SD)	48.5 (22.5)	36.7 (25.2)	0.017
Cardiovascular morbidity, no. (%)	11 (30.6)	31 (37.3)	0.34
Comorbid conditions, mean (SD)	0.86 (0.99)	0.83 (1.02)	0.88
Use of inhaled steroids, no (%)	29 (80.6)	64 (77.1)	0.81
**Symptoms, no. (%)**			
Dyspnoea	36 (100)	78 (93.9)	0.66
Cough	25 (69.4)	75 (90.4)	0.007
Expectoration	36 (100)	79 (95.2)	0.31
Grade of dyspnoea, mean (SD)	1.86 (0.83)	1.28 (0.73)	<0.001
**Exacerbations in the previous year, no. (%)**			
Number			0.32
≤2	20 (55.6)	58 (69.9)	
>2	16 (44.4)	25 (30.1)	
**Requiring hospital admission**			0.003
None	20 (55.6)	67 (80.7)	
≤1	11 (30.6)	15 (18.1)	
>1	5 (13.9)	1 (1.2)	
**Lung function tests, mean (SD)**			
FVC, mL	2936.9 (975.7)	2712 (927.6)	0.23
FVC, %	71.9 (21.0)	67.1 (18.4)	0.21
FEV_1_, mL	1423.9 (536.5)	1382 (443.1)	0.66
FEV_1_, %	47.3 (15.9)	45.8 (13.4)	0.61
FEV_1_/FVC	48.9 (11.2)	52.7 (13.5)	0.15
Sputum analysis			
Colour, mean (SD)	2.97 (0.94)	2.01 (1.08)	<0.001
**Pro-inflammatory cytokines, median (IQR) in pg/mL**			
IL-1, n = 53	47 (5-593)	29 (4-255)	0.14
IL-6, n = 53	134 (39-381)	169 (34-415)	0.18
IL-8, n = 61	8060 (460-31400)	4890 (201-15025)	0.09
TNF-alpha, n = 54	76 (11-269)	38 (11-71)	0.96

FVC = forced vital capacity; FEV_1 _= forced expiratory volume in the first second; IQR = interquartile range; IL= interleukin; TNF = tumour necrosis factor.

The results of the multivariate analysis were very similar when identifying the factors significantly associated with the presence of PPMs or on classifying the population according to bacterial load. In both cases, only the degree of dyspnoea and sputum colour were significantly and independently associated with the presence of PPMs and with high bacterial load. Sputum colour was a stronger indicator of the presence of positive cultures for PPMs than its load (Table 5).

**Table 5:** Results of multivariate analysis of factors associated with presence of bacteria in sputum and with high bacterial load.

Factor	OR	95% CI	*P *value
**Factors associated with bacteria in sputum**			
Degree of dyspnoea	2.63	1.53 - 5.09	0.004
Sputum colour	4.11	2.30 - 7.29	<0.001
**Factors associated with high bacterial load as opposed to no bacteria and low bacterial load**			
Degree of dyspnoea	2.01	1.17 - 3.46	0.012
Sputum colour	1.99	1.32 - 2.99	0.001

## Discussion

In the present study, bacterial colonisation of the airways by PPMs, mainly *H. influenzae *and *H. parainfluenzae*, was reported in 49% of patients with stable COPD. This finding adds evidence to a high prevalence of bacterial colonisation of airways in stable COPD reported by others [4,5,9-12]. Interestingly, our results using sputum samples are quite similar to those obtained in other studies with the use of the PSB technique or bronchial lavage for microbiologic assessment of the lower airways in COPD [4,5,11,12,20,21]. The possibility of sputum collection along a maximum of three monthly clinical visits and the use of the induced sputum technique in selected cases may have accounted for this high diagnostic yield of the sputum. However, most of our patients were able to produce a valid sputum sample for microbiological examination and induction of sputum was necessary in only 5 cases. A previous study by our group demonstrated that spontaneous and induced sputum yielded equivalent results in terms of frequency of bacterial colonisation and species recovered [22]. A pooled analysis of data from studies that used PSB demonstrated that a PPM load ≥10^2 ^CFU/mL should be considered abnormal and allowed the estimation that at least one quarter of the patients with stable COPD were colonised by PPMs [5]. Furthermore, most patients with exacerbated COPD had concentration of PPMs > 10^5 ^[4,5]. Since there is no universally accepted cut-off for high bacterial load in sputum samples, a 10^5 ^CFU/mL concentration was used in our study [4,8]. With this value, 30% of our total population and almost two thirds of the colonised patients in our study had a high PPM load.

Bacterial colonisation in our study was related to cumulative consumption of cigarette smoking, history of exacerbations in the previous year and sputum colour. Exacerbations in the previous year leading to hospitalisation were associated with increased bacterial load, although this relationship disappeared on multivariate analysis. In other studies, current smoking and severe airflow obstruction have been identified as predisposing factors for bacterial colonisation in stable COPD [11,12]. However, we did not observe significant differences in lung function between colonised and non-colonised patients. The relationship between lung function and frequency of colonisation is not clear, since a lack of association between FEV_1 _and colonisation has also been observed in other studies [8,12,21,23] and may be due, at least in part, to the under-representation of mild patients in most series as well as in the current study. Interestingly, the only two factors identified in multivariate analysis to be significantly and independently associated with both presence of bacterial colonisation and high bacterial load were a more severe degree of dyspnoea and a darker colour of sputum. The degree of dyspnoea is a marker of severity of COPD and being a categorical variable with a wider distribution in our population probably contributed to its demonstrated association with colonisation, in contrast to the severity of FEV_1 _impairment.

Regarding bronchial inflammation, it should be noted that we did not find increased sputum concentrations of pro-inflammatory cytokines in patients with bacterial colonisation. Different reasons may explain this finding, including a small number of patients with valid samples for analysis, the inter-individual variability in the sputum concentrations of the cytokines was very large [24], and there was a large number of patients with low bacterial loads. In fact, Hill *et al*. [8] have demonstrated that markers of inflammation increased progressively with increasing bacterial load in patients with stable COPD. Consequently, when our colonised patients were categorized according to high or low bacterial load, besides the persistence of the clinical differences already observed between the colonised and non-colonised groups (i.e., cigarette smoking, hospitalisations in the previous year, grade of dyspnoea and sputum colour) a non-significant trend towards higher sputum concentrations of inflammatory markers (except IL-6) was observed in patients with high bacterial load. Our results concur with previous observations regarding the lack of association between colonisation and increased IL-6 [9,10] but are discordant with other works showing significantly increased bronchial IL-8 and TNF-alpha in colonised patients, particularly with *H.influenzae *[9,10,21,23,25]. Therefore, our data, if confirmed in a larger sample of patients, would also suggest a dose-response relationship between bacterial load and bronchial inflammation and that a threshold of bacterial load might be necessary to elicit a significant inflammatory reaction in the airways [5,6,26]. In contrast, Sehti *et al*. [27] examined whether the increase in bacterial concentrations functions as a separate mechanism of exacerbation induction, independent of a new strain acquisition. In a prospective longitudinal cohort of COPD patients assessed during exacerbations and stable disease, sputum concentrations of pre-existing strains of *H. influenzae *and *H. haemolyticus *were not significantly different in exacerbation versus stable disease. Concentrations of *M. catarrhalis *and *S. pneumoniae *were even lower during exacerbations compared with stable periods. However, concentrations of new strains of *H. influenzae *and *M. catarrhalis *were increased during exacerbations, but the differences were small. These authors speculate that change in bacterial load was unlikely to be a major primary mechanism of exacerbation induction in COPD [27,28]. This hypothesis is a matter of debate, because the interpretation of what a significant increase in bacterial load is when measured in a logarithmic scale is not clear [10], and when transformed to a non-logarithmic scale, the differences in absolute bacterial counts were of a very high magnitude [29].

The identification of bronchial colonisation has clinical implications. Patel *et al*. [9] demonstrated that the presence of lower airway bacterial colonisation in stable COPD was significantly related to exacerbation frequency and severity. In the study of Rosell *et al*. [5], again high bacterial loads were associated with exacerbation and showed a statistically significant dose-response relationship between bacterial load and exacerbation after adjustment for covariates. In our study colonised patients had significantly more exacerbations and hospital admissions the year previous to the study compared with non-colonised patients, but the significance disappeared on multivariate analysis. It should be taken into account that our study was neither designed nor powered to demonstrate differences in exacerbation or hospitalisation rates between colonised and non-colonised COPD patients. Therefore, the identification of patients colonised by PPMs using a non-invasive and relatively inexpensive technique such as the analysis of sputum may play an important role in the management of severe and very severe COPD, particularly if intervention studies with antibiotics demonstrate improved clinical outcomes [13].

To facilitate the diagnosis of bronchial colonisation the use of a surrogate marker could be of interest. Purulence (colour) of sputum graded by the investigator with a simple scale from 1 to 5 revealed significant differences in colour between colonised and non-colonised patients. Patients with colour 3 or higher (dark yellow to green sputum) had a prevalence of bacterial colonisation greater than 80%. The relevance of sputum colour has been already described and validated for exacerbated patients in which yellowish or greenish sputum is significantly associated with a bacterial exacerbation compared with white (non-bacterial) sputum [30,31] but the relationship between sputum colour and bacterial colonisation in stable COPD has deserved little attention [8].

The present results should be interpreted taking into account some limitations of the study, particularly the small sample size may not have allowed determination of sputum concentrations of inflammatory markers in all samples, in most cases due to the small recovery of sputum that did not provide enough supernatant for the quantification of inflammatory mediators. The cross-sectional design did not allow the dynamics and time course of bacterial colonisation and airway inflammation during exacerbations to be examined. Patients with negative bronchodilator test were included to exclude individuals with asthma who are less likely to be colonised, but the results may not be extrapolated to partially reversible COPD patients. High concentrations of PPMs in sputum samples, however, is a simple parameter that may help to select candidates to participate in antibiotic trials of stable COPD in order to demonstrate bacterial eradication and potentially prolong time to exacerbation [6,32,33].

## Conclusions

Almost half of a population of ambulatory moderate to very severe COPD patients carry PPMs in their airways. Colonised patients had more severe dyspnoea, and sputum colour allows the identification of patients most likely to be colonised by PPMs.

## List of abbreviations

CFU: colony-forming units; CI: confidence interval; COPD: Chronic obstructive pulmonary disease; CT: computed tomography; FEV_1_: forced expiratory volume in one second; FVC: forced vital capacity; IQR: interquartile range; IL-1: interleukin-1; IL-6: interleukin-6; IL-8: interleukin-8; OR: odds ratio; PPMs: potentially pathogenic microorganisms; PSB: protected specimen brush; SD: standard deviation; TNF-alpha: tumour necrosis factor-alpha.

## Competing interests

Marc Miravitlles has received honoraria for consultancy and speaking at scientific meetings from Bayer Schering, GlaxoSmithKline, Boehringer Ingelheim and AstraZeneca. Cristian de la Roza is fully employed in the Medical Department of Bayer Schering Pharma. Antoni Torres has received honoraria for consultancy and speaking at scientific meetings from Bayer and Covidien. Alicia Marín, Eduard Monsó, Sara Vilà, Ramona Hervás, Cristina Esquinas, Marian García, Laura Millares and Josep Morera have no conflict of interest to disclose.

## Authors' contributions

MM designed the study, participated in the analysis and interpretation of data and wrote the manuscript. EM, JM and AT designed the study, and participated in the analysis and interpretation of data. AM and SV recruited the patients, collected data and participate in the design and analysis. CR participated in the design and analysis of the study. CE and RH collected and processed the samples, and created and cleaned the database. LM and MG perfomed the microbiological investigations. All authors read and approved the final manuscript.
